# Effect of a bioformulation with native *Bacillus subtilis* and *Bacillus thuringiensis* strains on the control of *Fusarium oxysporum* and the rhizosphere microbiome of strawberry (*Fragaria* × *ananassa*)

**DOI:** 10.3389/fmicb.2026.1932414

**Published:** 2026-09-04

**Authors:** Iván Darío Otero Ramírez, Sandra Magally Sánchez Trujillo, Iván Enrique Paz Narvaez, José Luis Hoyos Concha

**Affiliations:** Centro Internacional de Biotecnología Agroindustrial (CBA), Faculty of Agricultural Sciences, Universidad del Cauca, Popayán, Colombia

**Keywords:** *Bacillus subtilis*, *Bacillus thuringiensis*, bioinputs, *Fusarium oxysporum*, strawberry

## Abstract

**Introduction:**

Strawberry (*Fragaria × ananassa*) is an economically important fruit crop whose production has increased substantially in recent years. However, its productivity is severely affected by a wide range of pathogens, including *Fusarium oxysporum*, the causal agent of wilt. Although chemical control remains widely used, biological strategies have gained increasing attention because of their environmental benefits and contribution to sustainable agriculture. The objective of this study was to evaluate the biocontrol potential of native bacterial bioformulations against *F. oxysporum* in strawberry under field conditions.

**Methods:**

Bacteria isolated from rhizosphere soil and root samples collected from eight producing farms in Cauca, Colombia, were screened for antagonistic activity, and the most effective isolates were identified by 16S rRNA gene sequencing. Bioformulations based on *Bacillus subtilis* UCBF11 and *Bacillus thuringiensis* UCBF2 were developed as microbial “bioinputs” and evaluated under open-field conditions in strawberry crops inoculated with *F. oxysporum*. Agronomic variables such as root growth and biomass, biomass promotion indexes, and the bioinput efficiency index were determined. Additionally, the effect of the treatments on the rhizosphere microbiota was analyzed using high-throughput sequencing of the 16S rRNA and ITS regions.

**Results and Discussion:**

A total of 350 isolates were obtained. *B. subtilis* UCBF11 and *B. thuringiensis* UCBF2 showed high antagonistic activity against *F. oxysporum*, with inhibition percentages of 79.72% and 84.95%, respectively. Field application of these bioinputs significantly increased root length and biomass compared to the control treatments, with *B. subtilis* UCBF11 showing the most outstanding performance. Metataxonomic analysis showed modifications in the structure of bacterial and fungal communities among treatments, and bioinput application was associated with a lower increase in the relative abundance of *F. oxysporum*. These results demonstrate the potential of *B. subtilis* UCBF11 and *B. thuringiensis* UCBF2 as biological control agents against *F. oxysporum* in strawberry crops.

## 1 Introduction

Strawberry *Fragaria × ananassa* is one of the crops that has shown a significant increase in the fruit market in recent years, reaching a global production volume of approximately 10.7 million t by 2024, led by China, the United States, Egypt, and Mexico (FAO, 2025). In this context, Colombia contributed 110,401 t (AGRONET, 2025), where the department of Cauca, after Cundinamarca and Antioquia, ranked as the third-largest producer at the national level, contributing 16,629 t (15.06%). Within Cauca, municipalities such as Sotará (9,800 t), Puracé (3,432 t), Silvia (2,860 t), Bolívar (300 t), and Totoró (216 t) stand out (AGRONET, 2025).

The strawberry crop is affected by a wide range of phytopathogens, including fungi, bacteria, viruses, and nematodes, which impact various plant organs such as roots, crowns, leaves, and fruits (Dholi et al., 2023). Among these, soilborne diseases represent one of the most critical constraints in strawberry production systems, particularly those affecting the root system and crown, due to their persistence in the soil and their often latent development (Avilés et al., 2024). Root disease complexes are commonly associated with pathogens such as *Rhizoctonia* spp., *Pythium* spp., *Phytophthora* spp., *Verticillium* spp., and *Fusarium* spp., which may act individually or synergistically, leading to root necrosis, vascular dysfunction, and reduced plant vigor (Zhang et al., 2022; Zhang Y. et al., 2025).

Among soilborne pathogens, strawberry wilt caused by *Fusarium* spp. is one of the most detrimental fungal diseases, typically colonizing vascular tissues after entering through the roots and spreading to stems, petioles, and fruit stalks (Kumar et al., 2025). The primary agent, *Fusarium oxysporum* f. sp. *fragariae*, can infect strawberries from the nursery stage through flowering and fruiting. Upon invading through root wounds, the pathogen proliferates hyphae that induce the host to synthesize callose, colloids, and defensive compounds. These defense responses, combined with secreted toxins, obstruct vascular bundles and disrupt the transport of water and nutrients. Consequently, infection results in growth suppression, vascular browning in the crown, and the wilting and desiccation of older leaves, eventually resulting in plant death (Yang et al., 2024).

Strawberry disease management primarily integrates three strategies: cultural, chemical, and biological control. Cultural practices, such as crop rotation, soil solarization, and the use of resistant varieties, help mitigate pathogen pressure but often prove insufficient under high disease incidence (Lee et al., 2026). While chemical fungicides provide rapid efficacy, their intensive application has raised environmental concerns, pathogen resistance, and soil health degradation (Harba et al., 2020; Yang et al., 2024). Consequently, biological control has gained prominence as a sustainable alternative, utilizing beneficial microorganisms to suppress phytopathogens and improve overall plant health (Demir et al., 2023; Liu et al., 2025).

Within biological strategies, biological control agents (BCAs) have gained significant attention, primarily featuring bacterial genera such as Bacillus, Pseudomonas, and Streptomyces, as well as fungal agents like Trichoderma. Among these, *Bacillus* species are particularly noteworthy due to their resilience to fluctuating environmental conditions, including changes in pH, temperature, and osmotic stress (Menéndez-Cañamares et al., 2024). These microorganisms offer distinct advantages by secreting antimicrobial substances, effectively colonizing the soil, and contributing to soil health and microbial balance (Chen et al., 2026). Consequently, the use of *Bacillus* spp. has become a prominent research focus for controlling strawberry root rot and wilt caused by *Fusarium oxysporum* (Zhang Y. et al., 2025).

Furthermore, while the direct antagonistic mechanisms of BCAs are well documented and applying beneficial bacteria can counteract the growth of soilborne pathogens like *Fusarium* (Boulahouat et al., 2023; Rafiee et al., 2022), considerably less is known about how native bacterial bioinputs reshape resident soil microbial communities under open-field conditions, particularly in strawberry systems. This knowledge gap is especially evident in Colombia, where research has focused predominantly on agronomic productivity and conventional crop management. High-throughput sequencing and metataxonomic profiling now provide an opportunity to move beyond single-strain efficacy assessments toward a broader understanding of microbiome-level responses and rhizosphere ecological dynamics (Chen, 2026; Zhang C. et al., 2025).

To address this gap, this study evaluated the isolation, molecular characterization, and field performance of native antagonistic bacteria in a strawberry-producing region of Cauca, Colombia. It was hypothesized that native *Bacillus*-based bioinputs would modulate the soil metataxonomic profile by promoting beneficial bacterial groups, thereby mitigating the impact of *Fusarium* wilt on crop performance under field conditions. Accordingly, this study aimed to evaluate the biocontrol potential of native bacterial isolates against *Fusarium* spp. and assess their influence on soil microbial dynamics through 16 S rRNA-based metataxonomic profiling alongside key agronomic traits.

## 2 Materials and methods

### 2.1 Soil and root sampling in strawberry crops

Soil and root samples were obtained from strawberry crops in the Department of Cauca. Sampling was conducted across four production units in the municipality of Silvia [1- “El Paraíso” Farm (2°35’33.1”N, 76°21’01.6”W); 2- “Agua Salada” Farm (2°37’58.1”N, 76°20’42.6”W); 3- “El Fresal” Farm (2°37’54.8”N, 76°20’28.9”W); and 4- “Cacique Alto” Farm (2°39’17.208”N, 76°19’48.191”W)] and four production units in the municipality of Sotará [1- “La Prosperidad” Farm (2°12’51.6”N, 76°35’09.755”W); 2- “San Gabriel””Farm (2°16’11.058”N, 76°33’08.357”W); 3- “La Marena” Farm (2°16’39.797”N, 76°33’01.607”W); and 4- “San Gabriel 2” Farm (2°16’22.173”N, 76°3”’17.250”W)].

Samples were collected from healthy plants and plants with root disease symptoms (wilting, petiole necrosis, and/or root rot). For soil samples, a composite sampling of approximately 250 g was obtained from five plants; at each plant, a hole approximately 15 cm deep was dug near the root, and rhizosphere soil samples were collected. On the other hand, for root sample collection, a complete extraction of three plants with disease symptoms was performed, protecting their root system. All samples were transported under refrigeration using expanded polystyrene (EPS) boxes with gel packs to preserve their integrity until processing at the Centro Internacional de Biotecnología Agroindustrial (CBA) of the Universidad de Cauca.

### 2.2 Isolation and morphological characterization of rhizosphere bacteria

Bacteria were isolated using serial dilutions and the spread-plate method on Nutrient Agar (NA). From the collected soil samples, quartering was performed and 10 g were taken, which were placed in an Erlenmeyer flask with 90 mL of sterile distilled water (10^−1^ dilution). The mixture was agitated in a shaker at 120 rpm for 30 min, allowed to settle for 10 min, and then a 1 mL aliquot was transferred to a tube with 9 mL of sterile water (10^−2^ dilution). This procedure was repeated until a 10^−6^ dilution was reached. From each dilution, 100 μL were surface inoculated onto NA plates, distributed using glass beads, and incubated for 24 h at 30°C (Beyari and Alshammari, 2025).

Root samples were rinsed with distilled water, fragmented, and 10 g were collected, which were transferred to an Erlenmeyer flask with 90 mL of Nutrient Broth (NB) for pre-enrichment (24 h at 30°C and 100 rpm). After this time, serial dilutions and plating were performed as described above (Beyari and Alshammari, 2025).

The obtained isolates were grouped based on colony morphology, purified by the streak-plate method, and cryopreserved at -20 °C in 60% glycerol. Simultaneously, a macroscopic description of the colonies was performed considering shape, margin, elevation, surface, optical properties, size, configuration, and color. Furthermore, microscopic characteristics were established through Gram staining.

### 2.3 *In vitro* antagonism against *Fusarium oxysporum*

The *F. oxysporum* strain was obtained from the culture collection of the Aprovechamiento de Subproductos Agroindustriales (ASUBAGROIN) research group at the Universidad del Cauca. To perform the dual antagonism assay, *F. oxysporum* was activated by subculturing on potato dextrose agar (PDA) plates; the fungus was incubated for 5 days at 28°C.

For bacterial activation, bacterial strains were inoculated into tubes containing 9 mL of nutrient broth (NB) and incubated for 24 h at 30 °C. Subsequently, a 100 μL aliquot was transferred onto nutrient agar plates and spread uniformly across the entire surface using a T-shaped spreader. The plates were then incubated for 18 h at 30 °C to allow bacterial growth. After this period, agar disks approximately 0.5 cm in diameter containing bacterial biomass were obtained and used in the dual confrontation assay. Finally, for the dual confrontation, PDA plates were used, in which a disk of approximately 0.5 cm in diameter containing *F. oxysporum* was first inoculated; after 48 h of incubation at 28°C, a 0.5 cm diameter disk containing the bacteria to be evaluated was placed on the same PDA plate at a distance of 3 cm. All confrontations were performed in triplicate, and PDA plates inoculated with *F. oxysporum* without bacteria were used as controls (Hassan et al., 2023, Kim et al., 2024).

The plates containing the dual confrontation and the controls were incubated at 28°C for 7 days; after this time, the radial growth of the fungus was measured. With the data obtained, the percentage of growth inhibition of the pathogen was calculated using the following formula (Kim et al., 2024):


$$\%\mathrm{Inhibition}=\frac{C\,R\,G-C\,R\,T}{C\,R\,G}\times 100$$

Where: CRG is the radial growth of the control and CRT is the radial growth of the treatment.

### 2.4 Molecular identification of antagonistic strains

The bacteria that exhibited the highest inhibition percentages against *F. oxysporum* were identified by amplification of the 16S rRNA gene. For this purpose, DNA extraction was performed using the CTAB- solvent method and the obtained DNA was verified by 1% agarose gel electrophoresis. For the amplification of the 16S rRNA gene, the universal primers 27F (5′-AGAGTTTGATCCTGGCTCAG-3′) and 1492R (5′-GGTTACCTTGTTACGACTT-3′) were used; the quality of the amplicons was verified by 1% agarose gel electrophoresis, and they were sent for sequencing to Suministros Clínicos ISLA S.A.S. (Colombia). The obtained sequences were edited using BioEdit software (version 7.7.1) and compared with the NCBI database using the BLAST algorithm (Burbano et al., 2017; Li et al., 2021).

For the phylogenetic tree construction, the sequences of the identified strains were combined with reference sequences downloaded from the National Center for Biotechnology Information (NCBI) database, resulting in a single FASTA file. Subsequently, the file was imported into MEGA software, version 12.1.2 (Molecular Evolutionary Genetics Analysis), and a multiple sequence alignment was performed using the MUSCLE algorithm. The alignment was exported in MEGA format (.meg) and used to reconstruct the phylogenetic tree. Beforehand, the best-fitting nucleotide substitution model was selected using the Find Best DNA/Protein Models (ML) tool implemented in MEGA. The Hasegawa–Kishino–Yano model with invariant sites (HKY+I) was selected. Finally, the phylogenetic tree was reconstructed using the Maximum Likelihood method; ranch reliability was assessed using 1,000 bootstrap replicates, and the sequence of *Paenibacillus polymyxa* DSM36 was used as the outgroup.

### 2.5 Field trial design and bioinput application

Based on their high *in vitro* antagonistic activity, the selected bacterial strains were evaluated in a controlled field trial using strawberry seedlings (cv. Frontera) in the municipality of Sotará, Department of Cauca.

Prior to planting the strawberry seedlings, the soil was prepared by tilling. Cultivation beds were shaped with a width of 70 cm and a furrow spacing of 30 cm. Lime and organic matter were incorporated before finalizing the furrows. Finally, the beds were mulched with plastic film, planting holes were made at an approximate distance of 35 cm, and a drip irrigation system was installed.

The strawberry plant material used in this trial consisted of mother plants (cv. Frontera). Prior to planting, the producer’s traditional root-disinfection protocol was applied, which involved submerging the roots for 10 min in a 60 L solution containing 100 g of Zepelin 60 (fungicide), 2.5 g/L of Vitavax, and 40 mL of Agrodyne (bactericide). The disinfected plants were placed in baskets and subsequently transplanted into the experimental plots.

To produce the bioformulation, the bacterial strains were mass-cultured in nutrient broth supplemented with 10 g/L of glucose and harvested by centrifugation at 8,000 rpm for 10 min. The resulting biomass was mixed with talc carrier, dried for 12 h at 40 °C, and subsequently formulated by adding guar gum (2 g/L), powdered milk (4 g/L), and yeast extract (4 g/L).

On the other hand, *F. oxysporum* was activated on YGC agar plates. After 7 days of incubation at 25 °C, the fungal biomass present on the plates was transferred to 90 mL of distilled water supplemented with 0.01% Tween 80. The resulting suspension was used to inoculate flasks containing 100 g of rice prepared at a 1:1 ratio with distilled water and autoclaved at 121 °C for 20 min. The inoculated flasks were incubated for 5 days at 25 °C. Subsequently, the rice colonized by the fungus was removed and dried for 24 h at 35 °C. The dried mycelium was ground and mixed with guar gum (2 g/L), powdered milk (4 g/L), and yeast extract (4 g/L). Field monitoring was conducted over a 90-day period. The bioinputs were applied on days 5, 15, 25, 35, 50, 65, and 80, whereas *F. oxysporum* inoculations were performed on days 30 and 45. A volume of 100 mL of bioinput per plant was applied at a concentration of 2 g/L, prepared using the water available on the farm. The same procedure was used to inoculate *F. oxysporum*. To evaluate the developed bioformulations under field conditions, a randomized complete block design (RCBD) with three independent blocks was established. The experimental treatments consisted of three bioinputs (*Bacillus thuringiensis*, *Bacillus subtilis*, and a consortium/combination of both bacteria) and two controls (an uninoculated control (Ctrl) and a pathogen control inoculated only with *F. oxysporum*). The five treatments were distributed within each block.

Each experimental plot consisted of 24 plants, of which six plants at each end were used to minimize edge effects, while 10 plants located in the center of the plot were considered for the evaluation of agronomic and microbiological variables. To measure root length, two plants were collected from each experimental plot, corresponding to six plants per treatment. For the determination of root dry weight, one plant per experimental plot was used, corresponding to three plants per treatment. Disease severity was evaluated in 10 plants per block, corresponding to 30 plants per treatment. Finally, for the metataxonomic microbiological analysis, approximately 100 g of rhizosphere soil was collected from five randomly selected plants to form a composite sample.

### 2.6 Measurement of agronomic variables

The agronomic variables evaluated in this trial included root length, root dry weight, biomass promotion index, bioinput efficiency index, disease severity index, and disease reduction percentage.

Root length: On days 30 and 90 of the experiment, three plants per treatment were harvested, and the length of the newly developed roots was measured.

Root dry weight: On day 90, three plants per treatment were harvested. The roots were thoroughly washed with distilled water, sectioned, and oven-dried at 65 °C for 12 *h* until a constant weight was achieved. Finally, the root dry weight for each treatment was recorded (Kiani DehKian et al., 2024).

Biomass promotion index (BPI): This index determines the percentage increase in root biomass resulting from the applied treatments. It was calculated using the following formula:


$$B\,P\,I=\left(\frac{R\,T\,W-R\,C\,W}{R\,C\,W}\right)\times 100$$

Where: RTW is the root dry weight of the treatment, and RCW is the root dry weight of the reference control.

Bioinput efficiency index (BEI): This index evaluates the efficiency of the treatment in promoting root growth, expressing how many times the root biomass obtained with the bioinput exceeds that observed in the reference treatment. Its calculation was performed using the following formula:


$$B\,E\,I=\frac{R\,T\,W}{R\,C\,W}$$

Disease severity index percentage (%DSI): This index explains how affected the plants are by the disease. During the trial, it was measured on 10 plants per treatment, verifying the symptoms of each plant associated with root diseases, and scored according to the following scale: 1- healthy plant; 2- plants with black or brown spots on the petiole; 3- plants with wilting in 25% of the leaves; 4- plants with wilting in 50% of the leaves; 5- plants with wilting in 75% of the leaves; 6- plants with wilting in 100% of the leaves. With the data obtained, the disease severity index (%DSI) was calculated according to the following formula (Demir et al., 2023; Du and Li, 2024):


%DSI=(Σ⁢(g⁢r⁢a⁢d⁢e*#⁢o⁢f⁢p⁢l⁢a⁢n⁢t⁢s⁢i⁢n⁢t⁢h⁢a⁢t⁢g⁢r⁢a⁢d⁢e)M⁢a⁢x⁢i⁢m⁢u⁢m⁢g⁢r⁢a⁢d⁢e*T⁢o⁢t⁢a⁢l⁢#⁢o⁢f⁢e⁢v⁢a⁢l⁢u⁢a⁢t⁢e⁢d⁢p⁢l⁢a⁢n⁢t⁢s)x 100

Disease reduction percentage: This index expresses how much the progress of the disease was halted by the application of the. The calculation was performed according to the following formula (Demir et al., 2023; Du and Li, 2024):


%DR=((D⁢S⁢I⁢c⁢o⁢n⁢t⁢r⁢o⁢l-D⁢S⁢I⁢t⁢r⁢e⁢a⁢t⁢m⁢e⁢n⁢t)D⁢S⁢I⁢c⁢o⁢n⁢t⁢r⁢o⁢l)x 100

### 2.7 Soil metataxonomic sequencing and bioinformatics processing

On day 1 of the trial, prior to transplanting, six composite soil samples were collected from different points within the experimental plot. On days 30 and 45, composite rhizosphere soil samples were harvested from three randomly selected plants per treatment. In total, 36 samples were collected for metataxonomic analysis and transported under refrigeration to the CBA at the Universidad del Cauca, where they were stored at –80 °C until processing. Total DNA extraction from the soil samples was performed using the InviSorb^®^ Spin Soil DNA kit (INVITEK diagnostics).

Bacterial and fungal community profiling was performed by Macrogen Inc. (Seoul, Republic of Korea). For bacterial community analysis, the V3–V4 region of the 16S rRNA gene was amplified using the Illumina overhang adapter primers targeting the V3 (TCGTCGGCAGCGTCAGATGTGTATAAGAGACAGCCTACG GGNGGCWGCAG) and V4 (GTCTCGTGGGCTCGGA GATGTGTATAAGAGACAGGACTACHVGGGTATCTAATCC) regions. Fungal communities were characterized by amplification of the ITS region using the ITS3F (TCGTCGGCAGCGTCAGATGTGTATAAGAGACAGGCATCG ATGAAGAACGCAGC) and ITS4R (GTCTCGTGGGCTCGG AGATGTGTATAAGAGACAGTCCTCCGCTTATTGATATGC) primer pair with Illumina adapter overhang sequences. PCR amplifications were performed using Herculase II Fusion DNA Polymerase (Agilent Technologies, Santa Clara, CA, United States). Amplicons were purified using AMPure XP beads (Beckman Coulter, Brea, CA, United States), indexed with Nextera XT Index primers (Illumina, San Diego, CA, United States), and purified again before library quality assessment. Library concentration was determined using the PicoGreen assay on a VICTOR Nivo™ fluorometer (PerkinElmer, Waltham, MA, United States), and fragment size distribution was verified using the Agilent TapeStation D1000 ScreenTape system (Agilent Technologies, Waldbronn, Germany). Libraries were normalized, pooled, quantified by qPCR using the KAPA Library Quantification Kit (Roche/KAPA Biosystems), and sequenced on an Illumina MiSeq i100 platform using paired-end reads of 301 bp (2 × 301 bp).

Raw sequencing reads were analyzed on the Galaxy platform using QIIME2 tools. Paired-end sequences (R1 and R2) were quality-filtered, denoised, and merged into Amplicon Sequence Variants (ASVs) using the DADA2 algorithm via the *qiime dada2 denoise-paired* tool, adjusting the parameters to: Trunc_len_f = 250, Trunc_len_R = 220, MaxEE-f = 3, and MaxEE-r = 3, coupled with independent pooling and consensus-based chimera removal. Sequencing statistics, including the number of reads per sample, sequencing depth, and the number of amplicon sequence variants (ASVs), were obtained using the *qiime feature-table summarize* tool from QIIME2. Subsequently, low-frequency ASVs were filtered out; for bacterial data (16S), the filter parameters *p-min-frequency* = 20 and *p-min-samples* = 2 were applied, whereas a stricter filter was used for fungal sequences, retaining only ASVs with a minimum frequency of 500 reads per sample and present in at least two samples (*p-min-frequency* = 500; *p-min-samples* = 2).

Taxonomic assignment of bacterial ASVs was performed against the SILVA SSU Ref NR 99 database (version 138), while fungal taxonomic assignment was carried out using the UNITE sh_general_release_dynamic_19.02.2025 database, adjusting the parameters to *Trunc_len_f* = 0, *Trunc_len_R* = 0, *MaxEE forward* = 2, and *MaxEE reverse* = 3. The confidence threshold was 0.7 in both cases. To track the targeted microbial dynamics, the qiime taxa filter-table tool was employed to select the *Firmicutes*, considering that the active ingredients of the evaluated bioinputs were *B. thuringiensis* and *B. subtilis*. A filter for the order Hypocreales was also performed to monitor the behavior of *F. oxysporum* (Tenea and Reyes, 2024; Zhang C. et al., 2025).

## 3 Results

### 3.1 Bacterial isolation

The distribution of bacterial morphotypes obtained across the eight sampled farms is summarized in Table 1. Soil samples exhibited a higher number of isolates compared to root samples. Furthermore, the total number of isolates collected in each sampling area was highly similar, with 169 recorded in Silvia and 181 in Sotará.

**TABLE 1:** Bacterial morphotypes isolated across the different sampling farms.

Sample		Silvia					Sotará				
		F1	F2	F3	F4	Total	F5	F6	F7	F8	Total
Healthy plant	Soil	16	16	12	13	57	13	13	18	18	62
	Root	10	10	5	3	28	11	4	7	9	31
Diseased plant	Soil	23	15	10	12	60	14	12	16	22	64
	Root	12	5	5	2	24	12	2	9	1	24
Total		61	46	32	30	169	50	31	50	50	181

F1, El Paraíso; F2, Agua Salada; F3, El Fresal; F4, Cacique Alto; F5, La Prosperidad; F6, San Gabriel; F7, La Marena; F8, San Gabriel 2.

Regarding plant health status, 85 isolates were recovered from healthy plants and 84 from diseased plants in Silvia; in contrast, Sotará showed 88 isolates from diseased plants and 93 from healthy plants. Finally, the number of isolates per farm in Silvia was variable, with Farm 1 (El Paraíso) present the highest count at 61 isolates. Conversely, in Sotará, three production units showed identical counts of 50 isolates each (Table 1).

### 3.2 Selection of antagonistic bacteria against *Fusarium oxysporum*

**FIGURE 1:**
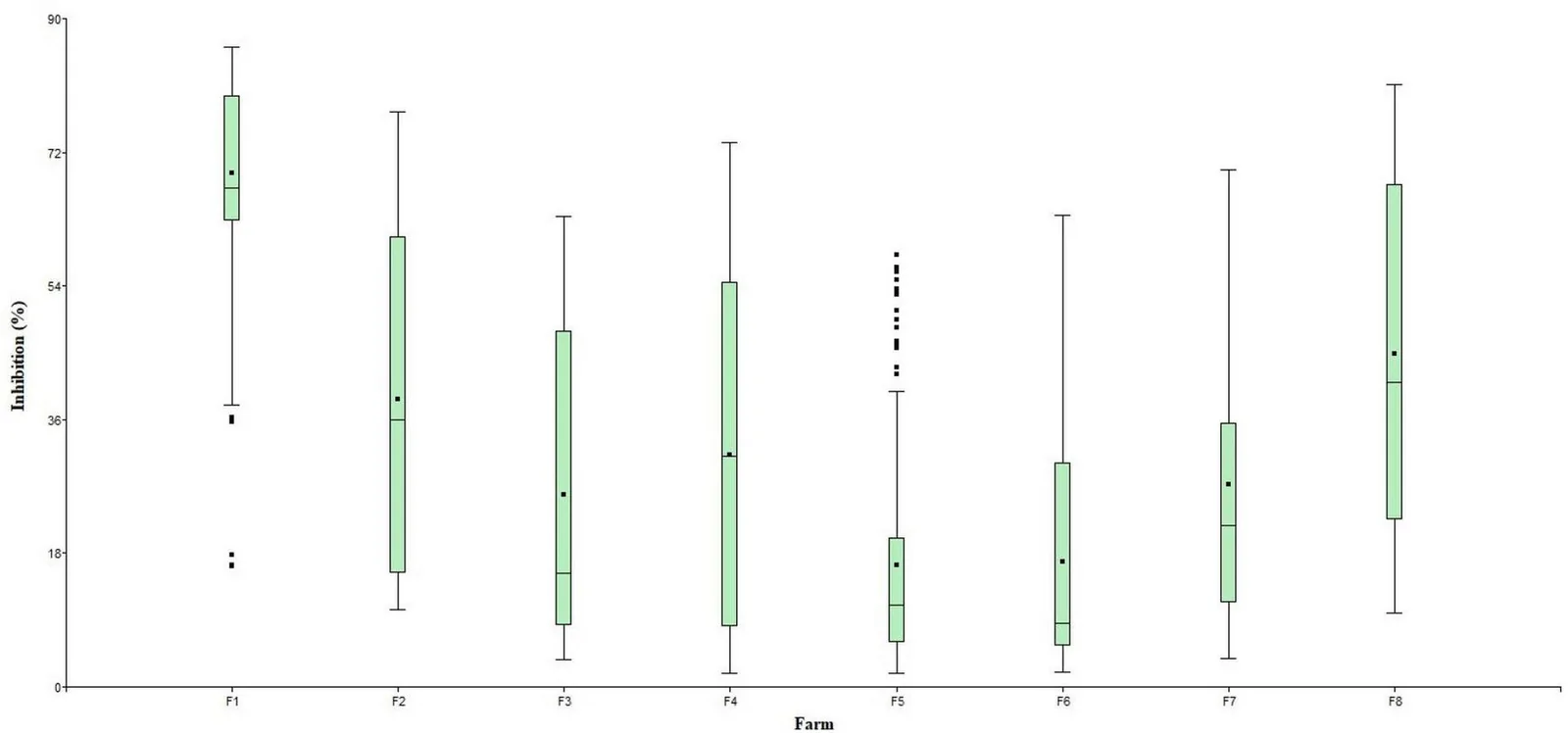
Antagonistic activity and distribution profile of bacterial isolates against *F. oxysporum* across different strawberry production farms in Cauca, Colombia. The central horizontal line inside the boxes represents the median; boxes correspond to the interquartile range (IQR, Q1–Q3); whiskers indicate data dispersion within 1.5 times the IQR, and individual points represent statistical outliers. F1, El Paraíso; F2, Agua Salada; F3, El Fresal; F4, Cacique Alto; F5, La Prosperidad; F6, San Gabriel; F7, La Marena; F8, San Gabriel 2.

The antagonistic response of the isolates obtained from the different farms against *F. oxysporum* is shown in Figure 1. In general, antagonistic isolates were found across all evaluated farms; however, inhibition percentages varied substantially, ranging from less than 10 to 86%. Farm 1 exhibited the isolates that recorded the highest inhibition, showing a distribution concentrated toward high antagonistic percentages. Farms 2 and 8 also presented high inhibition percentages but with a wider distribution. In contrast, Farms 5 and 6 reached the lowest percentages. Nonetheless, at least one isolate with an inhibition percentage greater than 50% was found on every farm.

The analysis of variance presented in Table 2 showed statistically significant differences (*p* < 0.0001) in pathogen inhibition percentages across the evaluated sampling farms. Tukey’s Honest Significant Difference (HSD) test separated the data into four distinct statistical groups, with Farm 1 exhibiting the highest inhibition percentages. The second group was comprised of Farms 8 and 2; the third group included Farms 4, 7, and 3; and finally, the fourth group consisted of Farms 6 and 5.

**TABLE 2:** Antagonistic activity of bacterial isolates obtained by farms.

Farm	Number of isolates	Mean Inhibition (%)	Minimum (%)	Maximum (%)
F1	61	69.19 ± 12.10^A^	16.28	86.20
F8	50	44.84 ± 23.10^B^	9.98	81.19
F2	46	38.76 ± 22.10^B^	10.35	77.51
F4	30	31.19 ± 240^C^	1.80	73.39
F7	50	27.22 ± 19.30^C^	3.87	69.65
F3	32	25.93 ± 20.40^C^	3.64	63.32
F6	31	16.77 ± 16.70^D^	1.95	63.57
F5	50	16.38 ± 15.20^D^	1.89	58.22

Values are expressed as mean ± standard deviation. Means followed by different letters within the column indicate statistically significant differences according to Tukey’s HSD test (*p* < 0.05).

Regarding the antagonistic response of the isolates relative to their isolation source (root or soil), no statistically significant differences were observed (*p* > 0.05). However, on average, the data presented in Table 3 indicate that isolates obtained from diseased soil matrices displayed the highest inhibition percentages; furthermore, at least one isolate with an antagonistic activity ranging between 80 and 86% was identified within each source category.

**TABLE 3:** Antagonistic activity of bacterial isolates obtained by isolation source.

Source	Number of isolates	Mean inhibition (%)	Minimum (%)	Maximum (%)
**Diseased root**	48	34.69 ± 23.45^A^	2.18	80.33
**Healthy root**	59	35.6 ± 25.13^A^	3.87	81.19
**Diseased soil**	124	37.89 ± 27.54^A^	1.89	86.20
**Healthy soil**	119	35.69 ± 25.75^A^	1.80	86.20

Means followed by the same letter within the column are not significantly different according to Tukey’s HSD test (*p* > 0.05).

Based on these results, five isolates with high antagonistic potential against *F. oxysporum* were selected. From Farm 1, isolates UCBF1, UCBF3, and UCBF4, all sourced from diseased soil, exhibited mean inhibition values of 82.63 ± 2.07%, 85.07 ± 0.78%, and 82.84 ± 3.57%, respectively. Likewise, isolate UCBF2, sourced from healthy soil on the same farm, recorded an inhibition of 84.95 ± 1.23%. Additionally, isolate UCBF11 from healthy roots on Farm 8 was selected with a mean inhibition of 79.72 ± 1.40%.

### 3.3 Molecular identification of antagonistic bacteria

The isolates selected for their superior antagonistic activity were identified by amplification and sequencing of the *16S rRNA* gene. Comparison of the obtained sequences with the NCBI database revealed that UCBF1, UCBF3, and UCBF4 correspond to *Bacillus nakamurai* (GenBank accession codes: PZ176915, PZ176917, and PZ176918, respectively). UCBF2, UCBF11 were identified as *Bacillus thuringiensis*, and *Bacillus subtilis*, respectively (GenBank accession codes: PZ176916, and PZ176919).

**FIGURE 2:**
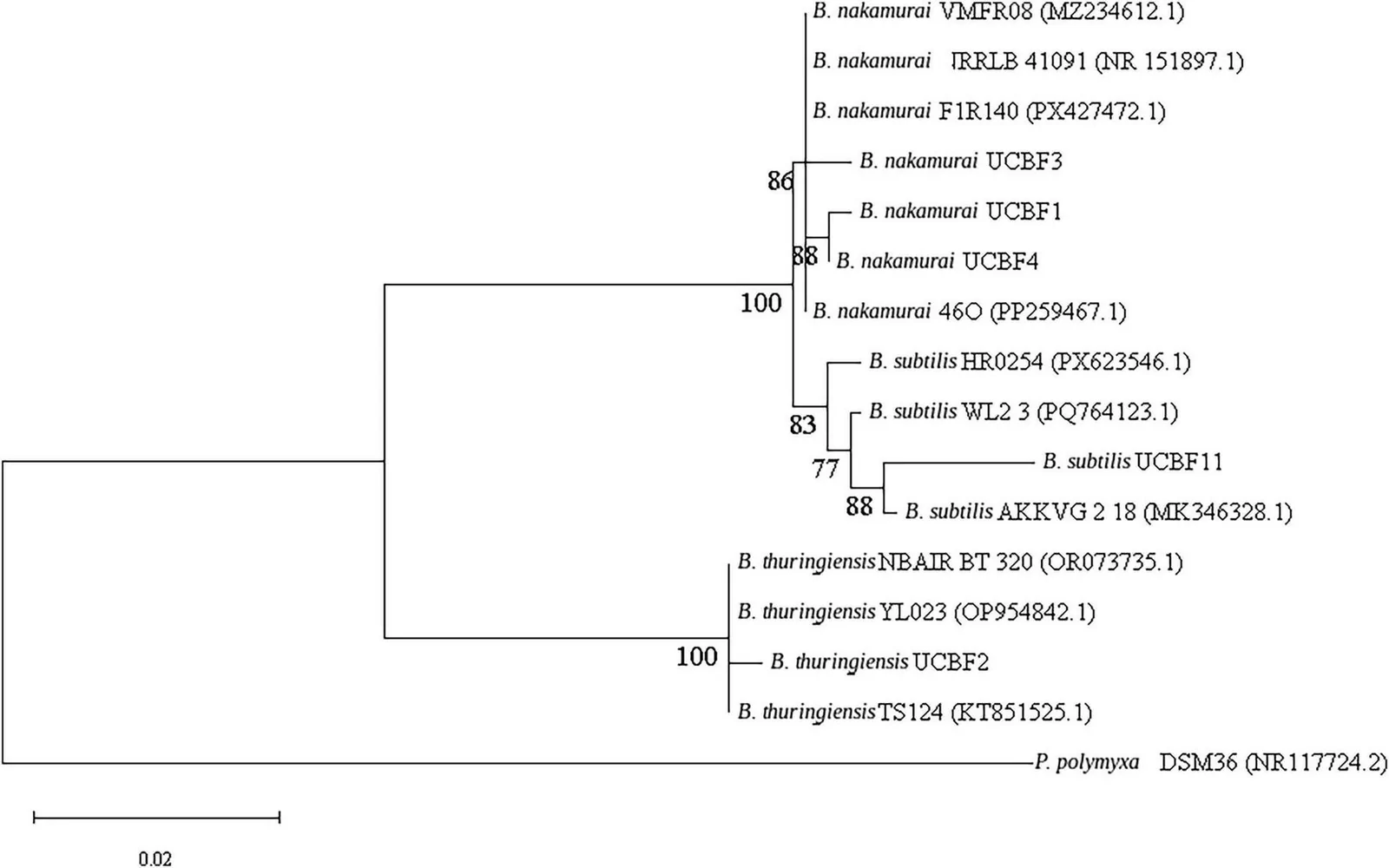
Phylogenetic tree based on the 16S rRNA gene showing the clustering of the selected bacterial isolates with reference *Bacillus* strains. The tree was constructed using the maximum likelihood method with the Hasegawa–Kishino–Yano model with invariant sites (HKY+I), with 1,000 bootstrap replicates. Isolate names correspond to the nomenclature used in the article.

Phylogenetic analysis based on the 16S rRNA gene sequences confirmed the taxonomic assignments obtained through BLAST. Three main clades were observed: one comprising *B. nakamurai*, which included strains UCBF1, UCBF3, and UCBF4; another comprising *B. subtilis*, which grouped strain UCBF11; and the third comprising *B. thuringiensis*, which included strain UCBF2 (Figure 2).

According to the results obtained, *B. nakamurai* UCBF3 exhibited the highest numerical inhibition percentage (85.07%), followed by *B. thuringiensis* UCBF2 (84.95%). However, the analysis of variance did not show statistically significant differences (*p* = 0.0544) among the five selected strains. Therefore, the final selection of strains for field evaluation considered both the *in vitro* inhibition percentage and technological characteristics related to the ease of obtaining bacterial biomass for incorporation into the formulation.

In this regard, during biomass production in liquid medium, the *B. nakamurai* strains produced visibly viscous biomass that was difficult to recover by centrifugation, thereby hindering the homogenization of the formulation. Although the cause of this behavior was not experimentally determined, it could be associated with the production of extracellular polymeric substances (EPS), which have been described in biofilm-forming *Bacillus* species (Netrusov et al., 2023). Future studies evaluating EPS production, biomass recovery efficiency, and alternative processing strategies will help confirm this hypothesis and more accurately determine the technological potential of these isolates for the development of bioinputs for strawberry cultivation. Therefore, the strains selected for field evaluation were *B. thuringiensis* UCBF2 and *B. subtilis* UCBF11

### 3.4 Controlled field trial

The *B. thuringiensis* UCBF2 and *B. subtilis* UCBF11 strains were used for the field trial.

#### 3.4.1 Root length

The root length measurements showed statistically significant differences among the evaluated treatments. Fisher’ multiple comparison test showed that the combined bioinput (*B. thuringiensis* UCBF2 and *B. subtilis* UCBF11) presented the highest root length values, reaching 2 cm and 11.67 cm at 30 and 90 days, respectively. In contrast, the control treatments without inoculum and those inoculated exclusively with *F. oxysporum* presented the lowest root length values (Table 4).

**TABLE 4:** Root length of strawberry plants subjected to different bacterial bioformulation treatments.

Treatment	30 days	90 days
	Mean (cm)	Mean (cm)
*B. thuringiensis* and *B. subtilis*	2^A^ ± 0.0	11.67^A^ ± 1.53
*B. thuringiensis* UCBF2	1.25^B^ ± 0.25	9^B^ ± 0
*B. subtilis* UCBF11	1.1^B^ ± 0.4	8.33^B^ ± 0.58
Control (without inoculum)	1^B^ ± 0.0	8^C^ ± 0B
*F. oxysporum*	1^B^ ± 0.0	6.33^C^± 1.15

Values represent the mean ± standard deviation. Means followed by different letters within the same column indicate statistically significant differences according to Fisher’s LSD test (*p* < 0.05).

#### 3.4.2 Root dry weight

The results for root dry weight, biomass promotion indices (BPI), and bioinput efficiency indices (BEI) are presented in Table 5. Regarding root dry weight, statistically significant differences were found among treatments (*p* < 0.0105). Fisher’s multiple comparison test grouped all treatments inoculated with bacterial bioinputs into the same category (group a), which recorded the highest root biomass values. In contrast, group b comprised control treatments without inoculum and those where *F. oxysporum* was applied alone, which exhibited lower root biomass values.

**TABLE 5:** Root dry weight and growth promotion indices of strawberry plants.

Treatment	Root dry weight (g)	* BPI-P	BPI-C	** BEI-P	BEI-C
***B. subtilis* UCBF11**	0.63^A^ ± 0.3	348.33^A^	151^A^	4.48^A^	2.51^A^
***B. thuringiensis* and *B. subtilis***	0.62^A^ ± 0.05	335.33^A^	144.33^A^	4.35^A^	2.44^A^
***B. thuringiensis* UCBF2**	0.57^A^ ± 0.1	292^A^	123^AB^	3.92^A^	2.23^AB^
**Control (without inoculum)**	0.26^B^ ± 0.03	81^B^	0A^BC^	1.81^B^	1^BC^
***F. oxysporum***	0.14^B^ ± 0.01	0^B^	-43.33^C^	1^B^	0.57^C^

*BPI, biomass promotion index; **BEI, bioinput efficiency index; P, values calculated relative to the pathogen-inoculated treatment; C, values calculated relative to the inoculated control treatment. Means followed by different letters within the same column indicate statistically significant differences according to Fisher’s LSD test (*p* < 0.05).

Similarly, the application of bioinputs was observed to improve the BPI. Although no statistical differences were found among the applied bioinputs, the treatment with *B. subtilis* UCBF11 stood out, achieving a root biomass increase of 348% relative to the pathogen-inoculated treatment and 151% compared to the control treatment without inoculum. Consistently, according to the BEI, this treatment increased root biomass up to 4.5 times compared to the pathogen-inoculated treatment and 2.5 times compared to the control.

#### 3.4.3 Disease severity index (%DSI) and reduction percentage

The analysis of variance (ANOVA) of the disease severity index (%DSI) in plants treated with the bioinputs showed statistically significant differences (*p* = 0.0003). Fisher’s multiple comparison test identified three distinct groups; the treatment where the pathogen was applied alone presented the highest disease severity, whereas the treatment with the *B. subtilis* UCBF11 bioinput resulted in the lowest severity values (Table 6).

**TABLE 6:** Disease severity index (%DSI) and disease reduction percentage (%DR) in strawberry plants.

Treatment	%DSI	Treatment	%DR-P	%DR-C
***F. oxysporum***	66^A^ ± 5.29	*B. subtilis* UCBF11	30.97^A^	20.32^A^
***B. thuringiensis* UCBF2**	58^B^ ± 2.00	*B. thuringiensis* and *B. subtilis*	29.89^A^	19.14^A^
**Control (without inoculum)**	57^B^ ± 3.00	Control (without inoculum)	13.49^B^	0^B^
***B. thuringiensis* and *B. subtilis***	46^C^ ± 3.46	*B. thuringiensis* UCBF2	11.57^B^	-2.06^B^
***B. subtilis* UCBF11**	45.33C ± 1.15	*F. oxysporum*	0^C^	-15.69^C^

%DSI, disease severity index percentage; %DR-P, disease reduction percentage relative to the pathogen-inoculated treatment; %DR-C, disease reduction percentage relative to uninoculated control. Means followed by different letters within the same column indicate statistically significant differences according to Fisher’s LSD test (*p* < 0.05).

Regarding the disease reduction percentage, the analysis of variance demonstrated significant differences among treatments. Fisher’s multiple comparison test differentiated three statistical groups, where the bioinputs formulated with *B. subtilis* UCBF11 and the combination of *B. subtilis* UCBF11 and *B. thuringiensis* UCBF2 registered the highest reduction values.

### 3.5 Metataxonomic analysis of the rhizosphere microbiome

#### 3.5.1 Core taxonomic composition (phylum, class, and order)

The bioinformatic analysis of the sequencing reads obtained from the strawberry cultivation soil showed that across the control treatments without inoculum (C0, C30, and C45) and the pathogen-inoculated control treatments (*F. oxysporum*: P0, P30, and P45). After quality control, denoising, and chimera removal using DADA2, 190.231 reads were obtained across 18 samples. The average sequencing depth was 10.568 reads per sample, and 6.666 ASVs were detected. Subsequently, after applying the low-frequency ASV filter, 64.020 reads were retained, with an average sequencing depth of 3.556 reads per sample and 734 ASVs. The raw sequencing data generated in this study were deposited in the Sequence Read Archive (SRA) of the NCBI under BioProject accession number PRJNA1502432.

**FIGURE 3:**
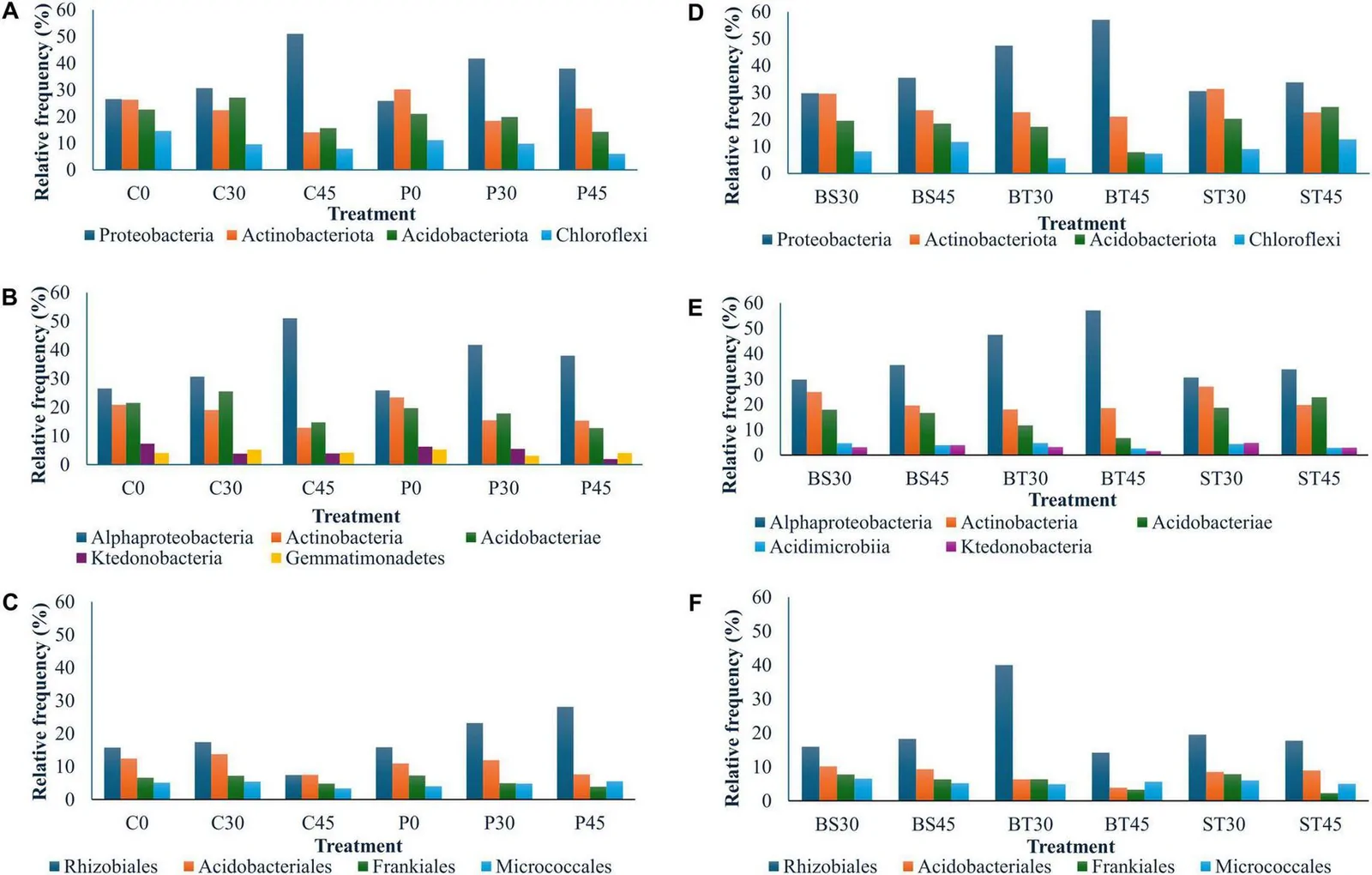
Metataxonomic analysis of soil bacterial communities in strawberry crops. Relative abundance profiles are displayed for: Phylum **(A,D)**, Class **(B,E)**, and Order **(C,F)** levels. Left panels **(A–C)** represent control groups: C (uninoculated control) and P (*F. oxysporum* pathogen control). Right panels **(D–F)** represent bioformulation delivery treatments: BS (*B. subtilis*), BT (*B. thuringiensis*), and ST (combined consortium of *B. subtilis* and *B. thuringiensis*). Numerical suffixes 0, 30, and 45 indicate the sampling days throughout the crop production cycle.

A total of 17 phyla, 27 classes, and 53 bacterial orders were differentiated. The most abundant phyla included Proteobacteria, Actinobacteriota, Acidobacteriota, and Chloroflexi. A trend toward an increase in the relative abundance of Proteobacteria from day 0 to day 45 was observed in both control treatments, while the remaining three dominant phyla decreased over time (Figure 3A).

At the class level, 27 taxonomic groups were differentiated, dominated by Alphaproteobacteria, Actinobacteria, Acidobacteriae, Ktedonobacteria, and Gemmatimonadetes. Among these, Alphaproteobacteria exhibited a progressive increase between days 0 and 45 in both the uninoculated control and the *F. oxysporum*-inoculated treatments (Figure 3B).

At the order level, 53 taxonomic groups were identified, with Rhizobiales, Acidobacteriales, Frankiales, and Micrococcales being the most prominent. Notably, the order Rhizobiales displayed a contrasting behavior; its relative abundance decreased from day 0 to day 45 in the uninoculated control treatment, whereas it increased from 15 to 28% in the treatment inoculated with *F. oxysporum*, suggesting a differential response of the native bacterial community potentially associated with the presence of the fungal pathogen (Figure 3C).

Regarding the metataxonomic profile of the rhizosphere soil treated with the bioformulations (*B. subtilis*, *B. thuringiensis*, and their combination), 200.006 reads distributed across 16 samples were obtained. The average sequencing depth was 12,500 reads per sample, and 7,310 ASVs were generated. Subsequently, after applying the low-frequency ASV filter, 53,436 reads were retained, with an average sequencing depth of 3,339 reads per sample and 618 ASVs.

Sixteen bacterial phyla were detected, revealing a taxonomic richness comparable to that of the control treatments. The most abundant phyla were Proteobacteria, Actinobacteriota, Acidobacteriota, and Chloroflexi. Proteobacteria showed an increase in relative abundance from days 30 to 45 across all bioinput treatments. Notably, the *B. thuringiensis* treatment achieved the highest relative frequency on day 45, exceeding the levels observed in the control treatments (Figure 3D).

At the class level, 28 taxonomic groups were identified, dominated by Alphaproteobacteria, Actinobacteria, Acidobacteriae, Acidimicrobia, and Ktedonobacteria. Similar to the controls, Alphaproteobacteria showed an increasing trend from days 30 to 45; however, the *B. thuringiensis* inoculation registered the highest relative frequency among all evaluated treatments (Figure 3E).

At the order level, 54 taxonomic groups were differentiated, with Rhizobiales, Acidobacteriales, Frankiales, and Micrococcales being the most abundant. The order Rhizobiales stood out due to its temporal variation across the evaluation periods. Specifically, at day 45, the relative abundance of Rhizobiales under the bioinput treatments was higher than that observed in uninoculated control, but lower than that of the *F. oxysporum* control. Furthermore, in the *B. thuringiensis* treatment, the relative abundance of Rhizobiales decreased sharply from 40% on day 30 to 14% at day 45, demonstrating a specific shifting response of this group driven by the bioformulation application (Figure 3F).

#### 3.5.2 Specific tracking of the phylum bacillota

The microorganisms utilized as active ingredients in the evaluated bioformulations belong to the genus *Bacillus*, family Bacillaceae, order Bacillales, class Bacilli, and phylum Bacillota (Firmicutes). In control treatments, this phylum represented a relative abundance of less than 1% of the total microbial community.

Analysis of the Firmicutes composition in the uninoculated control showed a predominance of the genus *Romboutsia*, followed by *Clostridium*, at the beginning of the experiment. At day 30, the native population of *Bacillus* increased by approximately 20%, whereas by day 45, *Clostridium* became the single dominant genus.

**FIGURE 4:**
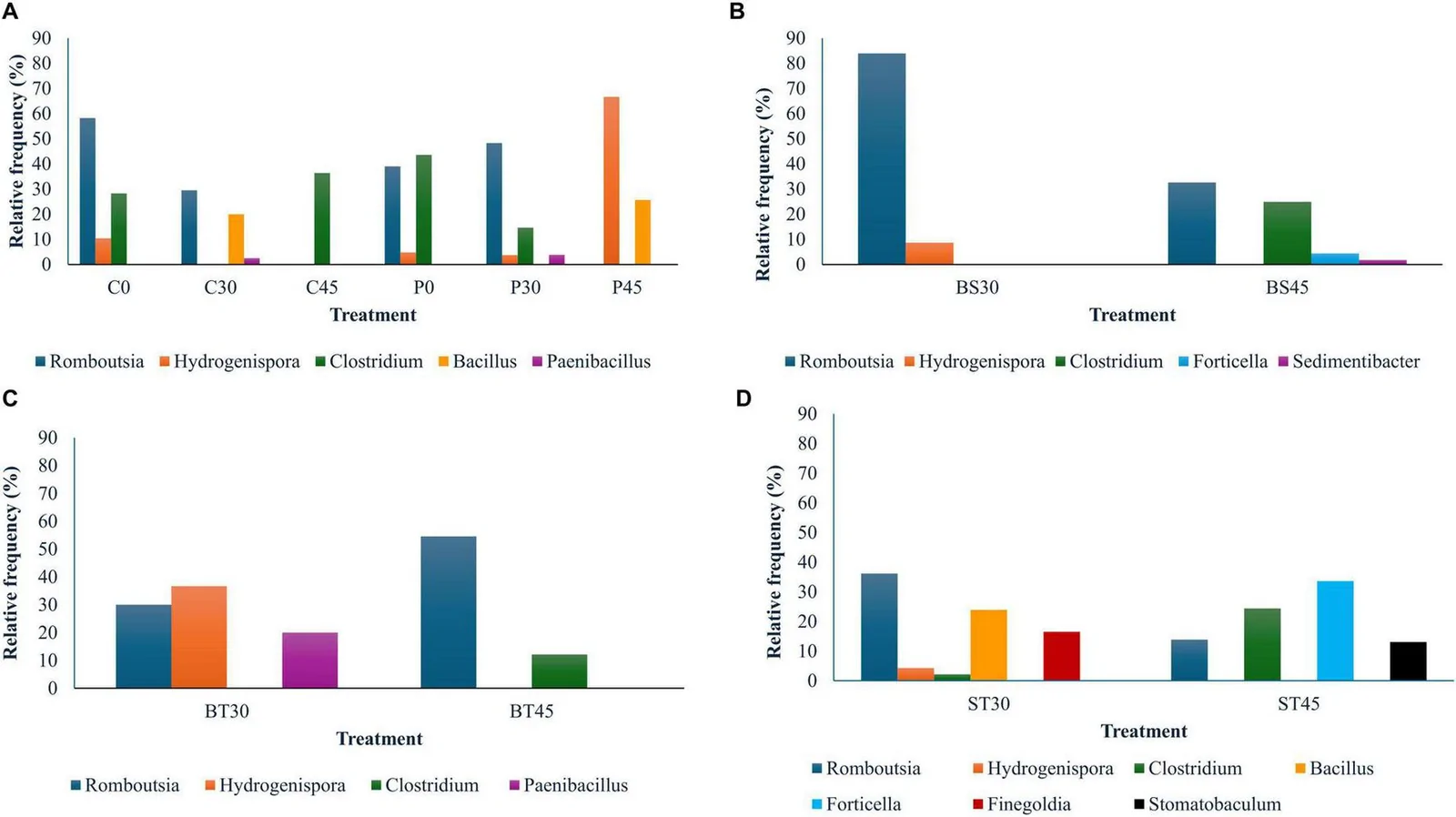
Metataxonomic tracking of targeted microbial dynamics within the strawberry rhizosphere microbiome. Relative abundance charts represent the structural shifts of the phylum *Bacillota* **(A)**, inoculation of *B. subtilis* **(B)**, inoculation of *Bacillus thuringiensis* **(C)**, and the combined microbial bioinput consortium **(D)**. C, uninoculated control; P, pathogen-inoculated control; BS, *B. subtilis* treatment; BT, *B. thuringiensis* treatment; ST, combined *B. subtilis* and *B. thuringiensis* treatment. Suffixes 0, 30, and 45 indicate sampling days.

In the control treatment inoculated with *F. oxysporum*, day 0 showed a composition pattern similar to the uninoculated control, characterized by the predominance of *Romboutsia* and *Clostridium*, though the latter registered a slightly higher relative abundance. However, at day 30, the relative abundance of *Clostridium* decreased, and by day 45, a clear shift in the community structure occurred, characterized by the dominance of *Hydrogenispora* along with a 20% relative abundance of *Bacillus*. The detection of *Bacillus* sequences in both control treatments confirms the presence of native populations of this genus residing within the strawberry rhizosphere (Figure 4A).

In the treatments inoculated with the *B. subtilis* bioformulation, a predominance of *Romboutsia* was recorded at day 30, which subsequently decreased at day 45 as the relative abundance of *Clostridium* increased. Additionally, genera such as *Forticella* and *Sedimentibacter*, which were completely absent in the control treatments, were detected (Figure 4B).

On the other hand, soil samples from the *B. thuringiensis* treatment exhibited a structural composition similar to the controls at early stages; however, the genera *Romboutsia* and *Clostridium* were strongly enriched at day 45, while *Hydrogenispora* decreased substantially (Figure 4C).

Finally, the bioinput containing the combination of *B. subtilis* and *B. thuringiensis* induced the most profound shifts within the Firmicutes community structure. At day 30, *Romboutsia*, *Bacillus*, and *Finegoldia* were detected; however, the latter two were no longer detectable by day 45. In contrast, a marked increase in the relative abundance of *Clostridium*, *Forticella*, and *Stomatobaculum* was observed at day 45 (Figure 4D)

#### 3.5.3 Structural dynamics of the order hypocreales

Regarding the fungal community present in the analyzed samples, the control treatments yielded 1,365,757 high-quality reads from 15 samples, with an average sequencing depth of 91,050 reads per sample and 4,451 ASVs. After filtering out low-frequency ASVs, 1,186,273 reads were retained, with an average sequencing depth of 79,084 reads per sample and 290 ASVs. In the bioinput treatments, 857,157 high-quality reads were obtained from 11 samples, with an average sequencing depth of 77,923 reads per sample and 3,365 identified ASVs. After filtering, 701,050 reads were retained, with an average sequencing depth of 63,731 reads per sample and 188 ASVs. Although the complete ITS dataset was processed for the taxonomic characterization of the fungal community, the analysis in this study focused on the order *Hypocreales*.

The phytopathogen *F. oxysporum* belongs to the family Nectriaceae, order Hypocreales, class Sordariomycetes, and phylum Ascomycota. Taxonomic analysis of the order Hypocreales revealed that *F. oxysporum* sequences were detected in the soil from the beginning of the experiment, indicating that *Fusarium* was already present in the field soil before the artificial inoculation

**FIGURE 5:**
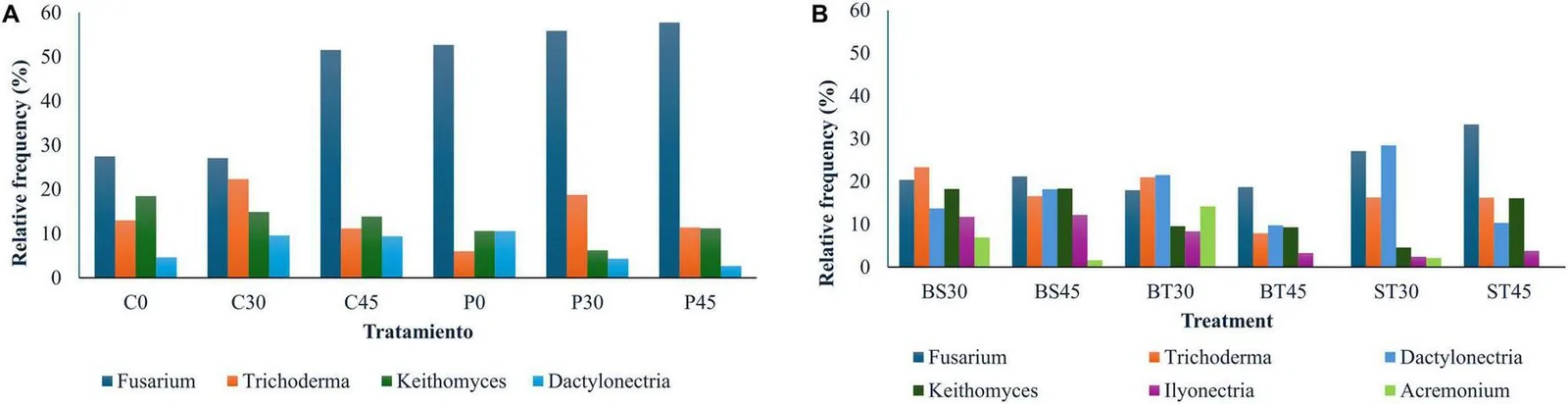
Fungal structural dynamics and relative abundance of the order *Hypocreales* in strawberry cultivation soils under biological and control management. Left panel **(A)** displays control frameworks: C (uninoculated control) and P (*F. oxysporum* pathogen control). Right panel **(B)** illustrates the tracking across bioformulated fields: BS, *B. subtilis* treatment; BT, *B. thuringiensis* treatment; ST, combined *B. subtilis* and *B. thuringiensis* treatment. Suffixes 0, 30, and 45 indicate sampling days.

In the control treatments, a severe increase in its relative abundance overtime was observed, reaching approximately 50% in the uninoculated control and approximately 58% in the treatment intentionally inoculated with *F. oxysporum*. In stark contrast, within the treatments managed with the bacterial bioformulations, the relative abundance of *F. oxysporum* remained restricted and never exceeded 33%. This finding suggests that the preventive application of the bacterial bioformulations was associated with a reduced increase in the relative abundance of *F. oxysporum* under field conditions. Additionally, until day 30, the population of the beneficial genus *Trichoderma* remained slightly higher than that of *F. oxysporum* across all bioinput treatments, with the sole exception of the combined *B. subtilis*–*B. thuringiensis* formulation (Figure 5).

The bioformulations were applied three times before pathogen inoculation to evaluate their performance as a preventive strategy under field conditions. This application schedule is consistent with the conventional practices of producers, who apply bioinputs before periods of high pathogen pressure to promote the establishment of beneficial microorganisms in the rhizosphere. In addition, the metataxonomic analyses detected *Fusarium* sequences in the rhizosphere from the beginning of the experiment, indicating that native populations of this genus were already present in the soil. Therefore, *Fusarium* inoculation was intended to increase pathogen pressure rather than introduce the pathogen into a pathogen-free system. Under these conditions, the lower increase in the relative abundance of *F. oxysporum* observed in the bioinput-treated plots, together with the lower disease severity, is consistent with a preventive biocontrol effect rather than a therapeutic response.

## 4 Discussion

### 4.1 Bacterial isolation, monoculture context, and *in vitro* antagonistic activity

The number of isolates obtained from a strawberry crop is variable and depends on factors such as sample origin, cultivation conditions, and the isolation methodologies employed. Authors such as Zhang et al. (2023) reported the isolation of 912 bacteria grouped into 192 species, asserting that a significantly higher number of isolates were found in the roots of diseased plants compared to healthy ones. Ning et al. (2025) isolated 113 bacterial strains from healthy strawberry plants and rhizosphere soils, while Chen et al. (2026) isolated 362 bacterial strains from the rhizosphere soil of strawberry plants. These results are consistent with the observations in this study, where 350 isolates were recovered, with soil being the primary source of isolation. In contrast to the findings reported by Zhang et al. (2023), the number of isolates obtained from healthy and diseased plants in the present study was similar, displaying a difference of less than 2%.

Strawberry (*Fragaria × ananassa* Duch.) is a fruit of great global interest due to its short growth cycle, considerable consumer demand, and high profitability (Zhang et al., 2023). These factors have encouraged the practice of continuous cropping or monoculture. In Colombia, specifically in the municipalities of Silvia and Sotará within the Department of Cauca where this study was conducted, it is common for growers to maintain strawberry crops for several years, thereby establishing a monoculture system.

Authors such as Su et al. (2022) note that continuous monoculture alters the natural balance of the soil by modifying its physical, chemical, and biological characteristics. Among the problems generated by this practice are low plant survival rates, weak growth, decreased productivity, and deterioration of fruit quality. At the soil level, it leads to the accumulation of toxic chemical substances (phenolic acids), a reduction in pH, changes in available NPK content, and modifications in the structure and diversity of microbial communities (Chen et al., 2020; Zhang C. et al., 2025). According to the results obtained in this study, 48.29% of the isolates originated from Silvia and 51.71% from Sotará. This similarity could be associated with the shared agronomic characteristics of continuous strawberry cultivation and the management practices implemented by growers. These practices have likely impacted the composition of the culturable microbiota present in the crop, resulting in a difference of less than 3.5% in the isolates obtained between the two municipalities. Nevertheless, complementary molecular analyses are required to further investigate this aspect.

According to Su et al. (2022), the impact on microbial diversity associated with continuous strawberry cultivation is of great importance, given that the structure and diversity of soil microbial communities play a crucial role in the equilibrium of this ecosystem and, consequently, in crop health. Beneficial microorganisms in the rhizosphere perform essential functions in nutrient acquisition, stress tolerance, host immune regulation, and protection against pests and diseases. In contrast, soilborne pathogens can cause root infections, thereby reducing yield and harvest quality. In this regard, Chen et al. (2026) state that if strawberries are continuously cultivated in the same soil, it favors the colonization of soilborne pathogens, such as *Fusarium* sp. Therefore, the identification of native microorganisms with antagonistic potential constitutes a relevant strategy for developing biological alternatives aimed at the sustainable management of diseases in strawberry crops.

The five strains selected for their high antagonistic activity against *F. oxysporum* under *in vitro* conditions belonged to the genus *Bacillus*. According to available literature, the genus *Bacillus* is a phenotypically and genotypically heterogeneous group of Gram-positive, endospore-forming bacteria, and it is one of the most widely distributed bacterial genera in terrestrial and agricultural environments. Furthermore, it has established itself as one of the most promising bacterial groups for biological control strategies (Rafiee et al., 2022).

Ning et al. (2025) mention that species of the genus *Bacillus* possess physiological characteristics that favor their adaptation to diverse environments, including endospore formation, low risk of resistance development, and higher stress tolerance, which confers a competitive advantage against pathogens. Among the mechanisms of action of *Bacillus*, competition for nutrients and space, production of antibiotics, enzymes, and siderophores, and/or the induction of systemic resistance (ISR) are prominent. Among the most recognized species are *B. velezensis*, *B. licheniformis*, *B. pumilus*, *B. amyloliquefaciens*, *B. cereus*, *B. subtilis*, and *B. thuringiensis* (Boulahouat et al., 2023). This aligns with the observations in this study, where the isolates with the highest antagonistic activity corresponded to strains of the genus *Bacillus*, particularly *B. subtilis*, *B. thuringiensis*, and *B. nakamurai*. However, the specific mechanisms underlying their antagonistic activity were not investigated and therefore cannot be inferred from the present results. Several studies have demonstrated the antagonistic capacity of *B. subtilis* against different soilborne pathogens, including *Verticillium* sp., *Penicillium digitatum*, and *Fusarium oxysporum* (Liu et al., 2021). In strawberry crops, Ning et al. (2025) reported that the *B. subtilis* TT-3 strain inhibited the growth of *F. oxysporum* by 69.68%, whereas Chen et al. (2026) and Tomar et al. (2026) recorded inhibition percentages of 72.94 and 73.23%, respectively. Similarly, Rafiee et al. (2022) reported *B. subtilis* XL62 as the strain presenting the best antifungal activity against *F. oxysporum* and *Rhizoctonia solani*. In comparison with these background studies, the *B. subtilis* UCBF11 strain evaluated in this study exhibited a higher inhibition percentage (79.72 ± 1.40%), highlighting its high potential as a biological control agent.

Regarding *B. thuringiensis*, it is known to be another species of the genus *Bacillus* characterized by the production of a wide variety of antimicrobial compounds with potential for pest and disease control. Additionally, various strains have been described as plant growth promoters, contributing to plant health and productivity. Traditionally, this species is associated with the biological control of insects through the production of Cry proteins, while its role as a fungal biocontrol agent remains poorly explored. However, previous studies have reported that some strains are capable of producing chitinases and lipopeptides, making them a promising biocontrol agent (Kiani DehKian et al., 2024).

Some studies have demonstrated the potential of *B. thuringiensis* to inhibit *Fusarium oxysporum* f. sp. *lycopersici* within a range of 40–50% (Beyari and Alshammari, 2025). In contrast, the wild strain *B. thuringiensis* UCBF2 isolated in this study achieved 84.95% inhibition of *F. oxysporum*. This result suggests that the wild strain UCBF2 possesses an elevated antagonistic potential under the experimental conditions evaluated. However, the mechanisms responsible for this activity were not investigated in the present study and should be addressed in future research possibly associated with a greater production of antifungal metabolites or superior competitive ability against the pathogen, a hypothesis that should be evaluated in subsequent studies.

Additionally, the literature review conducted revealed scarce information regarding the use of *B. thuringiensis* as a biological control agent against *F. oxysporum* in strawberry cultivation. In this sense, the results obtained expand the knowledge regarding the potential of this species for the biological management of soilborne pathogens in this crop and establish a precedent for future research oriented toward understanding its mechanisms of action and its performance under field conditions.

The dual-culture assays demonstrated that the selected strains significantly reduced the radial growth of *F. oxysporum* under *in vitro* conditions. Although several antagonistic mechanisms have been described for species of the genus *Bacillus* in previous studies, the present work did not evaluate the specific mechanisms responsible for the observed inhibition. Therefore, the antagonistic activity observed should be interpreted exclusively in terms of the reduction in fungal growth under the experimental conditions evaluated, while the underlying mechanisms remain to be investigated.

Finally, it is worth highlighting that obtaining native strains with high antagonistic capacity is of special importance, as these microorganisms are adapted to local edaphoclimatic conditions. This adaptation could favor their establishment and performance as biological control agents under the specific strawberry cultivation conditions of the Department of Cauca.

### 4.2 Rhizosphere microbiome dynamics and agronomic enhancement in strawberry

Regarding the bioinput application trial in the strawberry crop, authors such as Sangiorgio et al. (2022) mention that the plant and its microbiota are considered a holobiont, in which interactions between the host and associated microorganisms determine processes related to nutrient acquisition, plant growth, stress tolerance, and disease resistance. The composition of these communities responds to factors such as plant genotype, soil physicochemical properties, and implemented agricultural practices, including fertilization, pesticide use, and monoculture (Su et al., 2022; Wang et al., 2024). Consequently, understanding the structure of the rhizosphere microbiome and its response to bioinput application represents a key aspect for developing sustainable phytosanitary management strategies.

The metataxonomic analysis conducted in this study revealed a bacterial community composed of 17 phyla, 28 classes, and 54 orders. These values were lower than those reported by Su et al. (2022), Yang et al. (2023), and Wang et al. (2024), who found between 27 and 36 phyla, 62–113 classes, and more than 180 orders in strawberry crops. However, the dominant taxonomic composition was consistent; the phyla *Bacillota*, *Proteobacteria*, *Actinobacteriota*, *Acidobacteriota*, and *Chloroflexi* predominated, which have been described as core components of the rhizosphere microbiome of both healthy and diseased strawberry plants (Su et al., 2022; Wang et al., 2024; Yang et al., 2023). The differences in the total number of taxa are likely due to the methodologies applied regarding the sequencing process and bioinformatic processing criteria. Another influential factor includes the specific edaphoclimatic conditions of each study; nevertheless, it was demonstrated that the dominant taxonomic composition remains similar and aligns with reports for soil microbiota in strawberry cultivation.

Among the identified phyla, *Proteobacteria* exhibited the highest relative abundance across all treatments, which coincides with previous descriptions of the strawberry rhizosphere (Wang et al., 2024; Zhang et al., 2023). Because this group includes numerous bacteria associated with nutrient cycling, plant growth promotion, and disease suppression, its predominance could reflect high biological activity within the rhizosphere. In contrast, *Actinobacteriota* and *Acidobacteriota* showed a relative decrease toward the end of the experiment in most treatments. Authors such as Zhang et al. (2023) have pointed out that *Actinobacteriota* groups microorganisms that produce antibiotics and other bioactive metabolites capable of promoting plant growth and contributing to the suppression of soilborne diseases. However, despite the observed reduction, this phylum remained among the dominant groups in all treatments. This indicates that the application of bioinputs did not cause drastic alterations in the general structure of the bacterial community, but rather adjustments in the relative abundance of specific groups.

Furthermore, regarding the behavior of the phylum *Bacillota*, to which the strains used as the active ingredients of the bioinputs belong: despite periodic applications of *B. subtilis* and *B. thuringiensis*, this phylum accounted for less than 1% of the total bacterial community throughout the experiment. This behavior partially coincides with reports by Zhang et al. (2023) and Wang et al. (2024), who observed a decrease in *Bacillota* in plants affected by Fusarium wilt. However, genus-level analysis revealed significant changes in the composition of this phylum, including variations in *Bacillus*, *Romboutsia*, *Clostridium*, *Forticella*, and *Sedimentibacter*, among others.

These results indicate that the effect of the bioinputs was not associated with a marked increase in the relative abundance of *Bacillus* within the bacterial community. Instead, the observed taxonomic shifts may reflect changes in the composition of specific bacterial groups. Whether these changes represent an ecological reorganization associated with disease suppression remains a hypothesis that requires validation through functional approaches, such as metagenomic or metatranscriptomic analyses, together with the evaluation of genes involved in microbial interactions.

It is important to highlight that the bioinputs were applied from the establishment of the trial and periodically thereafter during crop development, whereas experimental inoculation with *F. oxysporum* was performed on day 30. Furthermore, metataxonomic analysis showed that *F. oxysporum* was already part of the soil fungal community from the beginning of the experiment. In this context, the observed changes in microbial composition are likely a response to initial colonization by the pathogen and the continuous application of bioinputs. In this regard, authors such as Zhang C. et al. (2025) mention the “cry for help” theory, which proposes that plants under biotic stress modify the composition of their root exudates to recruit beneficial microorganisms and restrict pathogen development.

This interpretation also helps explain the changes observed within the fungal community. Despite the presence of *F. oxysporum* in all samples from the beginning of the experiment, the application of bioinputs limited the increase in its relative abundance after experimental inoculation. While the pathogen reached abundances close to 50–58% in the control treatments, its abundance did not exceed approximately 33% in the bioinput treatments. Concurrently, an increase in the genus *Trichoderma* was observed during the first 30 days in most bioinput-treated groups, a behavior that could be related to competitive exclusion processes or modifications in the rhizosphere environment induced by the inoculated strains.

This study does not establish causal relationships among these microbial groups. Nevertheless, the results suggest that the reduction in the relative abundance of *F. oxysporum* occurred concurrently with changes in the composition of the bacterial and fungal communities following the application of the bioinputs. Whether these taxonomic shifts reflect a functional ecological reorganization that contributes to pathogen suppression remains to be determined and should be evaluated using functional approaches, such as metagenomic and metatranscriptomic analyses, as well as analyses of genes involved in microbial interactions. Various authors have noted that *F. oxysporum* infection profoundly modifies the structure of bacterial and fungal communities due, among other factors, to the production of metabolites such as fusaric acid, which can alter microbial interactions and affect plant development (Yang et al., 2023). In this study, changes were observed in the phyla *Bacillota* and *Ascomycota* (specifically within the *Hypocreales* order) after pathogen inoculation. These results support the current concept of biological management based on microbiome modulation, where the objective is not solely to eliminate the pathogen, but rather to favor the establishment of functional microbial communities capable of increasing the resilience of the soil-plant system.

In addition to the modifications observed in the microbiome, the application of bioinputs produced favorable effects on root development and disease behavior. Treatments with *B. subtilis* UCBF11 and *B. thuringiensis* UCBF2 significantly increased root length and dry biomass compared to the controls, with *B. subtilis* UCBF11 showing the most prominent performance. These results were consistent with the bioinput efficiency index and the reduction in the disease severity index, indicating that the effect of the selected strains transcended the biological control of *Fusarium*. Various studies have demonstrated that species of the genus *Bacillus* promote plant growth through the production of phytohormones, siderophores, volatile compounds, nutrient solubilization, and the induction of systemic resistance (ISR). Consequently, the greater root system development observed likely increased water and nutrient absorption capacity and contributed, along with microbiome modulation, to limiting disease progression (Boulahouat et al., 2023). However, these mechanisms were not evaluated in the present study. Thus, although greater root development was observed in the bioinput treatments, the physiological processes underlying this response remain to be elucidated. The present work integrated the isolation of native strains, bioformulation development, agronomic evaluation, and metataxonomic analysis of the microbial community. This approach allowed linking the continuous application of *B. subtilis* UCBF11 and *B. thuringiensis* UCBF2 to modifications in the rhizosphere microbiome structure, a lower relative abundance of *F. oxysporum*, an improved root system response, and a reduction in disease severity. Taken together, these results suggest that the efficacy of bioinputs formulated with native strains does not depend exclusively on their antagonistic capacity against the pathogen, but also on their ability to modulate the taxonomic composition and ecological interactions of the rhizosphere microbiome, thereby influencing soil–plant system dynamics following bioinput application. Nevertheless, the functional significance of these changes remains hypothetical and should be investigated through complementary functional approaches.

## 5 Conclusion

The obtained isolates allowed the selection of native *Bacillus subtilis* and *Bacillus thuringiensis* strains with high antagonistic capacity against *Fusarium oxysporum* under both laboratory and open-field conditions. The bioinputs formulated with these strains favored root system development, reduced disease severity, and were associated with changes in the taxonomic composition of the rhizosphere microbiome, together with a lower relative abundance of *F. oxysporum*. Collectively, these results demonstrate the potential of the evaluated native strains as biological control agents for strawberry cultivation under Colombian conditions.

Although the observed changes in the rhizosphere microbiome coincided with improved plant performance and reduced disease severity, the present study does not establish causal relationships between these responses. Therefore, the functional significance of the observed taxonomic shifts and their contribution to pathogen suppression should be considered hypotheses requiring validation through complementary approaches, such as metagenomic and metatranscriptomic analyses or targeted functional gene analyses.

## Data Availability

The datasets presented in this study can be found in online repositories. The names of the repository/repositories and accession number(s) can be found at: https://www.ncbi.nlm.nih.gov/genbank/, PZ176915; https://www.ncbi.nlm.nih.gov/genbank/, PZ176917; https://www.ncbi.nlm.nih.gov/genbank/, PZ176918; https://www.ncbi.nlm.nih.gov/genbank/, PZ176916; https://www.ncbi.nlm.nih.gov/genbank/, PZ176919; https://www.ncbi.nlm.nih.gov/genbank/, PZ176920; https://www.ncbi.nlm.nih.gov/, PRJNA1502432.
